# Cotargeting of CYP-19 (aromatase) and emerging, pivotal signalling pathways in metastatic breast cancer

**DOI:** 10.1038/bjc.2016.405

**Published:** 2016-12-06

**Authors:** Stine Daldorff, Randi Margit Ruud Mathiesen, Olav Erich Yri, Hilde Presterud Ødegård, Jürgen Geisler

**Affiliations:** 1 Department of Oncology, Akershus University Hospital (AHUS), Lørenskog N-1478, Norway; 2 Institute of Clinical Medicine, University of Oslo, Campus AHUS, Oslo N-0313, Norway

**Keywords:** breast cancer, aromatase inhibitor, mTOR inhibitor, PI3K inhibitor, CDK4/6 inhibitor, anastrozole, letrozole, exemestane

## Abstract

Aromatase inhibition is one of the cornerstones of modern endocrine therapy of oestrogen receptor-positive (ER+) metastatic breast cancer (MBC). The nonsteroidal aromatase inhibitors anastrozole and letrozole, as well as the steroidal aromatase inactivator exemestane, are the preferred drugs and established worldwide in all clinical phases of the disease. However, although many patients suffering from MBC experience an initial stabilisation of their metastatic burden, drug resistance and disease progression occur frequently, following in general only a few months on treatment. Extensive translational research during the past two decades has elucidated the major pathways contributing to endocrine resistance and paved the way for clinical studies investigating the efficacy of novel drug combinations involving aromatase inhibitors and emerging drugable targets like mTOR, PI3K and CDK4/6. The present review summarises the basic research that provided the rationale for new drug combinations involving aromatase inhibitors and the main findings of pivotal clinical trials that have already started to change our way to treat hormone-sensitive MBC. The challenging situation of oestrogen receptor-positive and human epidermal growth factor receptor 2-positive (HER2+) MBC is also shortly reviewed to underline the complexity of the clinical scenario in the heterogeneous subgroups of hormone receptor-positive breast cancer patients and the increasing need for personalised medicine. Finally, we summarise some of the promising findings made with the combination of aromatase inhibitors with other potent endocrine treatment options like fulvestrant, a selective oestrogen receptor downregulator.

Following several decades with antioestrogen dominance concerning first line-therapy in oestrogen receptor-positive (ER+) metastatic breast cancer (MBC), aromatase inhibitors (AIs) of the third generation (letrozole, anastrozole, exemestane) became the preferred drugs in this setting approximately two decades ago (Geisler and Lønning, 2005). The pivotal trials comparing tamoxifen with letrozole, anastrozole or exemestane as first-line therapy in the metastatic setting are summarised elsewhere and are not given in details here (Gibson *et al*, 2009). Meanwhile, AIs of the third generation have been established as one of the preferred choices in all clinical phases of ER+ MBC as well as in the neoadjuvant and adjuvant setting for selected groups of patients.

Many patients with ER+ MBC experience an initial stabilisation or even regression of their metastatic burden during first-line therapy with an AI. However, in the majority of patients, endocrine resistance and disease progression occurs frequently, following only a few months on therapy. Thus, intensive research during the past two decades has elucidated several mechanisms contributing to tumour adaptation during aromatase inhibition and oestrogen suppression (Dowsett *et al*, 2005; Ma *et al*, 2015). Some of the signalling pathways involved in the development of endocrine resistance include targetable molecules like the kinase mammalian target of rapamycin (mTOR), the phosphatidylinositol-3-kinase (PI3K) and the cyclin-dependent kinases 4 and 6 (CDK4/6). Development of novel drugs targeting key players in the signalling intracellular cascades paved the way for new studies testing these novel compounds given in combination with AIs compared with more traditional AI monotherapy.

The aim of the present paper is to summarise the experience made hitherto with the most promising new drugs that have proven clinical efficacy in combinations with AIs, as well as to review novel agents that are currently tested in ongoing early-phase clinical trials. The challenging situation of ER+ and human epidermal growth factor receptor 2-positive (HER2+) MBC is also shortly reviewed to underline the complexity of the clinical scenario in the heterogeneous subgroups of ER+ breast cancer patients and the increasing need for personalised medicine.

## Aromatase inhibitors *vs* aromatase inactivators

**Figure 1:**
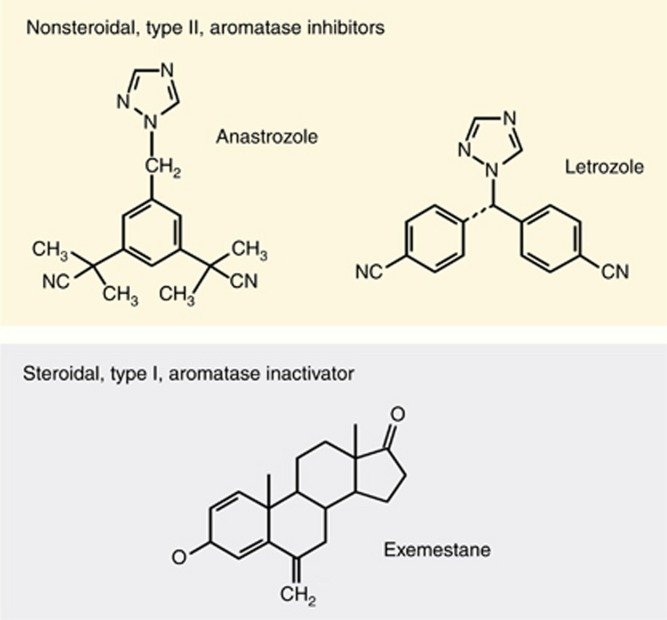
**Chemical structures of anastrozole, letrozole and exemestane.**

Nonsteroidal AIs act as competitive, reversible CYP-19/aromatase inhibitors, whereas steroidal AIs act as irreversible (‘suicide') inactivators of aromatase, causing decomposing of the aromatase molecule after binding to the inactivator (Hong and Chen, 2006). Many compounds with aromatase inhibitory effects have been tested in MBC patients during the past three decades (Geisler, 2003). Meanwhile, the orally administered compounds belonging to the third generation are currently used worldwide in the majority of countries. The drugs are classified as either nonsteroidal AIs (triazoles like anastrozole and letrozole) or steroidal aromatase inactivators (exemestane) because of their molecular structures and mode of action (Figure 1). Importantly, a lack of cross-resistance has been documented between these two major groups of aromatase inhibitory compounds, allowing sequential use in the metastatic setting (Bertelli *et al*, 2005; Lønning, 2009; Beresford *et al*, 2011; Van Asten *et al*, 2014). However, the precise mechanisms explaining the clinically observed lack of cross-resistance have not been fully clarified yet. Some of the possible explanations for this important clinical phenomenon have been discussed elsewhere (Lønning, 2009).

## Mechanisms of resistance to aromatase inhibitors

Breast cancer tumours are highly heterogeneous showing early subclonal development because of a variety of mutations and activated pathways that are only incompletely understood (Yates *et al*, 2015). So far, we know that most of the tumour cells become resistant to AIs after exposure for a given time (secondary or acquired resistance). However, primary or ‘*de novo*' resistance towards AIs has been reported to occur in up to 30% of ER+ MBC patients (Ma *et al*, 2015). An important goal of ongoing research is therefore to identify these nonresponding patients before therapy or early during therapy in order to either avoid nonfunctional therapies or to adapt treatment to the underlying tumour biology from the start. To predict the latter and at the same time find out how we may treat women who experience disease progression on AI treatment, we need to investigate the individual mechanisms that are responsible for endocrine resistance in a particular patient. In the following chapters we will briefly summarise some of the best documented mechanisms that are likely to cause resistance to AIs in breast cancer patients, as these mechanisms guided the way for the development of potentially meaningful new drug combinations.

**Figure 2:**
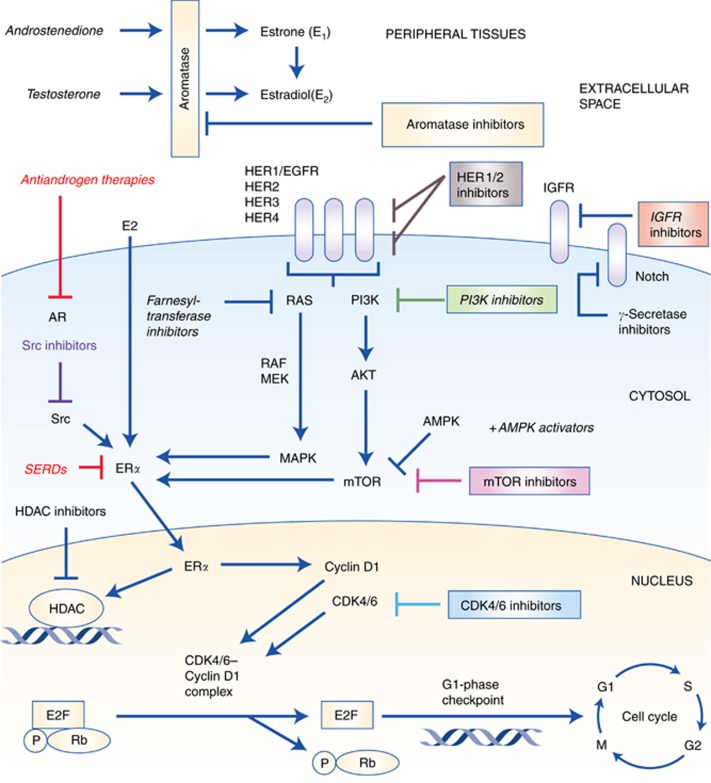
**Pivotal intracellular signalling cascades involved in endocrine resistance and site of action for selected targeted anticancer drugs.** AMPK=adenosine monophosphate-activated protein kinase; AR=androgen receptor; CDK4/6=cyclin-dependent kinase 4/6; E2=oestradiol; E2F=E2F transcription factors; ER*α*=oestrogen receptor-*α*; HDAC=histone deacetylase; HER2=human epidermal growth factor receptor-2; IGFR=insulin like growth factor receptor; P=phosphate; Rb=retinoblastoma protein; MEK=mitogen-activated ERK-activating kinase; mTOR=mammalian target of rapamycin; SERD=selective oestrogen receptor downregulator.

Activation of growth factor receptors (GFRs) and bidirectional crosstalk between the epidermal growth factor receptor family (EGFR/HER1, HER2, HER3, HER4) and ER*α* (Figure 2) has been associated with both *de novo* and acquired resistance to AIs (Gee *et al*, 2005; Lupien *et al*, 2010). Other growth factors besides the EGFR family are the insulin-like growth factor receptor-1 (IGFR-1), fibroblast growth factor receptor 1 (FGFR1) and their downstream signalling pathways, including the PI3K/Akt and the mitogen-activated protein kinase (MAPK) pathway (Liu *et al*, 2014; Ma *et al*, 2015). Activation of these tyrosine kinases may induce downstream effects like phosphorylation of ER*α* and alterations in the recruitment of its co-regulators. Overexpression of GFR signalling through EGFR or HER2 may also lead to activation of MAPK in ER+ breast cancer, causing loss of ER*α* expression (Ma *et al*, 2015). Primary significant overexpression of HER2 has been observed in as many as 10–15% of MBC and is associated with an increased metastatic potential, decreased disease-free and overall survival (OS) rates as well as early onset of endocrine resistance (Fan *et al*, 2015). Increased HER2 expression has been seen in sequential tumour biopsies from breast cancer patients after initiation of neoadjuvant AI therapy (Flågeng *et al*, 2009). This may indicate that AI therapy somehow triggers the expression of HER2, representing one of the many mechanisms causing acquired resistance. Additional inhibition of the HER2 pathway may reactivate ER*α* and sensitise the tumour to antihormonal therapies (Ma *et al*, 2015).

The PI3K/Akt/mTOR pathway (Figure 2) is another pivotal and frequently altered signalling pathway in human breast cancer. Studies have shown that at least 20–50% of all breast cancer tumours harbour mutations in PI3K (Sanchez *et al*, 2011; Lopez-Knowles *et al*, 2014), with mutations of the catalytic subunit of PI3K (PIK3CA) being the most common (Miller *et al*, 2011; Geuna *et al*, 2015). There is a growing body of evidence suggesting that alterations of PI3K play a crucial part in the development of acquired resistance towards AIs by regulating the ER*α* expression (Ma *et al*, 2015). This has caused considerable efforts to synthesise selective PI3K inhibitors for clinical use (Bendell *et al*, 2012; Liu *et al*, 2013; Ndubaku *et al*, 2013; Ando *et al*, 2014; Mayer *et al*, 2014; Rodon *et al*, 2014; James *et al*, 2015; Sarker *et al*, 2015; Wang *et al*, 2015; Massacesi *et al*, 2016). Further downstream along this pathway, we find an additional drug target, the kinase mTOR, that is essential to several intracellular signalling pathways promoting cell proliferation, differentiation, metabolism, migration and survival (Alberts *et al*, 2015; Geuna *et al*, 2015). The mTOR may be activated from aberrant upstream signalling or may be altered itself. Mutation of a subunit in the mTOR1 complex may phosphorylate and thereby activate the function domain 1 of the ER (Geuna *et al*, 2015). Beyond mutations that involve activation of PI3K and mTOR specifically, the entire PI3K/Akt/mTOR pathway may become abnormally activated following, for example, loss of PTEN (the pivotal negative regulator of the PI3K pathway), loss of inositol polyphosphate-4-phosphatase or mutations of Akt1 (Ma *et al*, 2015).

Altered ER signalling may also cause ligand-independent gene transcriptions and cell proliferation (Thomas and Gustafsson, 2015). Thus, it is thought to be one of the crucial mechanisms involved in endocrine resistance in general and in resistance during AI therapy in particular. The ER mutations most commonly cluster in the carboxy-terminal ligand-binding domain of oestrogen receptor 1 (ESR1), with Y537S, C or N and D538G being the most common. These mutations are rare in treatment-naive breast cancer, but more common in MBC, ranging from 11% to 55% (Ma *et al*, 2015). The incidence of ESR1 mutations seems to increase in patients who have a progressive disease following several endocrine treatment lines including AIs. Mechanisms such as translocations, amplifications, point mutations or aberrant expression or mutations of ER co-regulators have been described (Clarke *et al*, 2015; Ma *et al*, 2015).

In addition, active androgen receptor (AR) signalling has also been suggested to contribute to endocrine resistance during therapy with nonsteroidal AIs (Rechoum *et al*, 2014). Although the AR is expressed in 50–70% of all breast cancers, it is reported to be positive in 80–90% of all ER+ BC (Park *et al*, 2010). The AR positivity and overexpression have been linked to HER2 stimulation and activation of other signalling pathways by transcriptional upregulation of AR-dependent genes (Fujii *et al*, 2014; O'Shaughnessy *et al*, 2016). The AR negativity has also been shown to predict early treatment failure during adjuvant AI therapy (Elebro *et al*, 2015). As a consequence, subgroups of breast cancer patients may benefit from treatment with antiandrogens or selective AR modulators.

Aberrant expressions of molecules involved in the cell cycle machinery have recently been associated with endocrine resistance as well (Rocca *et al*, 2014). The CDKs play an important role in the control of check points during the cell cycle (Figure 2). Their levels rise and fall in a cyclic manner throughout the cell cycle, leading to cyclical phosphorylation and dephosphorylation of intracellular proteins that regulate important steps in the cell cycle. Of the 13 CDKs described in human cells, cyclin D is particularly important in the late G1 phase by binding CDK4/6 and thereby inducing the synthesis of cyclin E. This leads to the formation of the CDK2–cyclin E complex that in turn contributes to cell cycle progression through the restriction point and into the S phase of the cell cycle (Vidula and Rugo, 2016). Several signalling cascades induce the expression of cyclin D: Ras/Raf/MAPK, NF-*κ*B, Jak/STAT and steroid hormones (Alberts *et al*, 2015). The mechanism of action of the CDK4/6–cyclin D complex is phosphorylating and thus inactivating the tumour suppressor gene retinoblastoma (Rb) and its related proteins. When phosphorylated, the Rb protein relieves the inhibition of the transcription factor E2F (Figure 2), a promoter of genetic sequences encoding products that mediate S-phase entry and mitosis (Dickson, 2014; Rocca *et al*, 2014). Typical mechanisms involved in endocrine resistance at this stage are loss or phosphorylation of the Rb protein, deletion of the CDK4/6-inhibitor p16 or amplification of CDKs (Carey and Perou, 2015). Downregulation of the Rb pathway in ER+ breast cancer seems also to be associated with a more aggressive tumour growth and rapid recurrence on endocrine therapy (Cadoo *et al*, 2014; Witkiewicz and Knudsen, 2014). Loss of CDK4/6 cell cycle regulation occurs in 20–35% of breast cancer and promotes CDK2 activity, driving the cell cycle progression without the need of CDK4/6 (Vidula and Rugo, 2016). As a consequence, specific CDK4/6 inhibitors like palbociclib have no activity in Rb-deficient cells (Cadoo *et al*, 2014). The majority of ER+ breast cancer cells, however, do have intact Rb and it is therefore urgent to identify other clinically useful predictive biomarkers before treatment with CDK4/6 inhibitors (Carey and Perou, 2015).

Finally, numerous noncoding RNAs (ncRNA) have recently been shown to play a part in breast cancer progression and acquired endocrine resistance. MicroRNA (miRNA) and long non-coding RNA (lncRNA) are small molecules involved in the regulation of gene expression via translational transcription or degradation of mRNA transcripts. Overexpression of miRNAs and lncRNAs are associated with aberrant signalling and unregulated survival of cancer cells. More detailed information covering the contribution of miRNAs and so on during the development of endocrine resistance have been summarised in recent overviews and are not given here in detail (Hayes and Lewis-Wambi, 2015).

## Novel strategies to delay drug resistance during aromatase inhibitor therapy in mbc

### Aromatase inhibitors given in concert with drugs targeting HER1 (EGFR) and/or HER2 in MBC

One of the first strategies widely tested in clinical trials in this respect was the combination of epidermal growth factor receptor-1 (EGFR/HER1) targeting compounds and AIs (Leary and Dowsett, 2006). The European Organisation for Research and Treatment of Cancer (EORTC) recently published a randomised, multicentre phase II study comparing anastrozole monotherapy with the combination including the EGFR-inhibitor gefitinib (Tryfonidis *et al*, 2016). Disappointingly, gefitinib did not improve progression-free survival (PFS) when added to anastrozole. To the contrary, a phase II study published in 2010 compared the same combination and could show an improvement of ∼6-month PFS in the combination arm (Cristofanilli *et al*, 2010). However, both studies included ER+ MBC patients without defining the EGFR (mutational) status as an inclusion criterion, resulting in minor subgroups of enrolled patients actually being EGFR mutation positive. Interestingly, EGFR T790M mutations have recently been observed in biopsies of completely untreated primary breast cancer, indicating immediate drug resistance against most of the available EGFR targeting drugs in subgroups of MBC patients (Bemanian *et al*, 2015).

**Figure 3:**
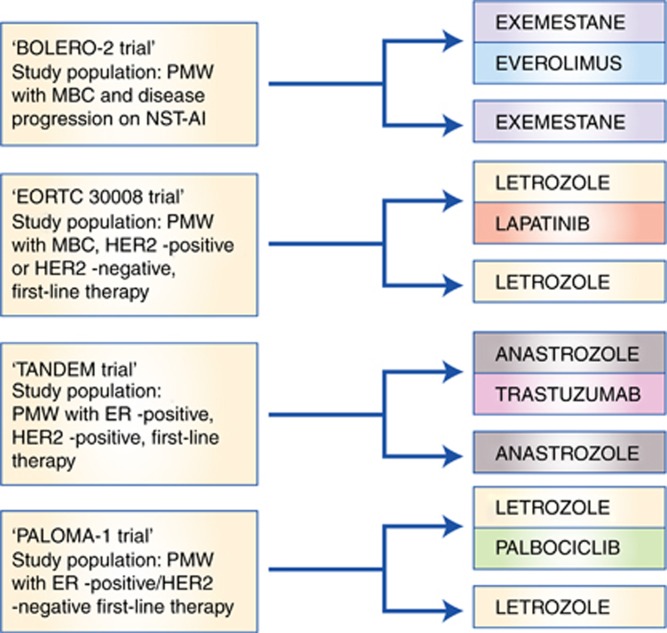
**Design of important clinical trials comparing aromatase inhibitors alone *vs* aromatase inhibitors given in combinations with novel targeted therapies.** MBC=metastatic breast cancer; NST-AI=nonsteroidal aromatase inhibitor; PMW=postmenopausal women.

In contrast, simultaneous use of HER2 targeting drugs and AIs has been challenged in a few pivotal trials that have already changed the standard care for ER+/HER2+ stage IV breast cancer patients (Riemsma *et al*, 2012). We mention here only two pivotal phase III trials in detail. The randomised, placebo-controlled TANDEM trial (Figure 3) showed that adding trastuzumab, a recombinant IgG1 monoclonal HER2 antibody against HER2, to anastrozole significantly improved the outcome in ER+/HER2+ MBC (Kaufman *et al*, 2009). Briefly, 207 postmenopausal ER+/HER2+ patients with MBC were included and randomly assigned to receive either anastrozole with trastuzumab or single-agent anastrozole. Patients in the combination arm experienced significantly improved mean PFS of 4.8 months (compared with 2.4 months in the monotherapy group, *P*=0.0016). There were more grade 3 and 4 adverse events in the combination arm, but all side effects were reversible and none were life threatening. The second major phase III trial was published by Johnston *et al* (2009; EORTC 30008; Figure 3), where letrozole was combined with lapatinib, an orally active dual HER1/HER2 inhibitor, that works by inhibiting the domains of the intracellular tyrosine kinases of both EGFR/HER1 and HER2 receptors (Paul *et al*, 2008). The results revealed that a combined strategy of lapatinib and letrozole significantly increased PFS in patients with ER+/HER2+ MBC (Schwartzberg *et al*, 2010). A total of 1286 ER+ patients were enrolled in the study and randomly assigned to receive either letrozole and lapatinib or letrozole and placebo. Approximately 17% of patients in each arm were HER2+. In the HER2+ subgroup (*n*=219), PFS significantly increased from a mean of 3.0 to 8.2 months (*P*=0.019). Importantly, there was no improvement in PFS in the ER+/ HER2− subgroup when lapatinib was added to letrozole. Finally, in preclinical models, Curley *et al* (2015) showed that HER3 signalling mediates resistance to letrozole, suggesting that MBC patients expressing HER3 may benefit from adding a specific ERBB3 (HER3) inhibitor such as the anti-ERBB3 antibody seribantumab to antihormonal therapy.

In summary, both trastuzumab and lapatinib have been established as targeting drugs that should be combined with traditional AIs in selected, ER+/HER2+ patients. Meanwhile, ongoing trials are testing a variety of novel combinations of aromatase inhibitors and anti-HER2 targeting drugs given in concert with mTOR inhibitors or CDK4/6 inhibitors.

### Aromatase inhibitors given in combination with mTOR inhibitors

Two mTOR inhibitors have been tested so far in combination with AIs: temsirolimus and everolimus. Both compounds unfold their action by binding to FKBP12, a protein receptor on the mTOR complex 1 (mTORC1) (Klumpen *et al*, 2010), a complex that has the ability to phosphorylate and thereby activate the function domain 1 of the ER. By inactivating mTORC1, mTOR inhibitors block the activation of the ER. Combining mTOR inhibitors with AIs has therefore been suggested to be a promising strategy to delay the onset of AI resistance (LoRusso, 2013). The pivotal BOLERO-2 trial (Table 1 and Figure 3), a randomised multicentre, phase III trial, included 724 postmenopausal women with ER+ MBC who had recurrence or progression on nonsteroidal AI treatment in the adjuvant or metastatic setting (Baselga *et al*, 2012). Patients were randomly assigned in a 2 : 1 ratio, the largest group receiving the mTOR inhibitor everolimus in combination with the steroidal AI exemestane. The BOLERO-2 trial showed significantly longer PFS in the combination group compared with the exemestane monotherapy group (10.6 *vs* 4.1 months). However, a follow-up publication 2 years later showed no statistically significant improvement of OS (Piccart *et al*, 2014). Following publication of the BOLERO-2 results, the combination of exemestane and everolimus was established as a standard combination in patients progressing on monotherapy with nonsteroidal AIs in many countries. The HORIZON study, a randomised, phase III, placebo-controlled study, tested the efficacy and safety of letrozole in combination with the mTOR inhibitor temsirolimus *vs* letrozole in monotherapy (Wolff *et al*, 2013). A total of 1112 patients were enrolled. Surprisingly, there was no overall improvement in PFS in the combination arm. However, an exploratory analysis showed significantly longer PFS in patients below the age of 65 years (9.0 *vs* 5.6 months; Wolff *et al*, 2013).

### Aromatase inhibitors given in combination with PI3K inhibitors

The PI3K mutations occur in up to 50% of ER+ MBC. Some of these mutations leave the enzyme constitutively active (Figure 2) and thereby a drugable target (Sanchez *et al*, 2011; Geuna *et al*, 2015). Preclinical models have showed that inhibition of the PI3K/mTOR pathway decreases tumour activity and induced apoptosis when combined with oestrogen deprivation in ER+ tumour cells (Sanchez *et al*, 2011), providing a rationale for combining the two regimens in a clinical setting. Both pan-PI3K and the PI3K*α*-selective inhibitors have been developed, but only phase I–II clinical trials have been published so far (Table 1). Mayer *et al* (2014) published a phase Ib trial in 2014, involving 51 postmenopausal women with histologically confirmed ER+/HER2− MBC. All patients received continuously letrozole as the basic endocrine therapy. In addition, all participants were randomised to either continuous or intermittent administration of the pan-PI3K inhibitor buparlisib. Maximum tolerated dose (MTD) in both treatment arms was 100 mg day^−1^, and the combination of letrozole and buparlisib was all in all well tolerated. Other dose-escalation studies with buparlisib show that 100 mg day^−1^ was a well-tolerated recommended dose for use in future studies (Bendell *et al*, 2012; Ando *et al*, 2014; Rodon *et al*, 2014). Common drug-related adverse events were hyperglycaemia, nausea, diarrhoea, fatigue, transaminitis, mood disorders and rash (Bendell *et al*, 2012). Several other PI3K inhibitors (such as pictilisib, pilarasib, voxtalisib and idelalisib) are currently tested in clinical trials (Liu *et al*, 2013; Ndubaku *et al*, 2013; Blackwell *et al*, 2015; James *et al*, 2015; Sarker *et al*, 2015). Although the latter has just recently been approved by the US Food and Drug Administration (FDA) for various haematological malignancies, we are currently waiting for crucial data from ongoing phase II–III trials to define the role of PI3K inhibitors as additional therapy to AIs.

### Combinations of aromatase inhibitors and CDK4/6 inhibitors

The CDKs play an important role in the cell cycle checkpoints by interacting with the E2F/Rb complex (Figure 2). Following phosphorylation by the cyclin D1–CDK4/6 complex, the transcription factor E2F is released from Rb, leaving E2F available for initiating the transcription of genetic products necessary for entering the S phase of the cell cycle. Without the cyclin D1–CDK4/6 complex, E2F remains bound to Rb, and the cell does not proceed to the G1 phase. The recently FDA-approved, potent and highly selective inhibitor of CDK4/6, palbociclib, represents one of the new promising drugs prohibiting cell cycle progression (Rocca *et al*, 2014; Beaver *et al*, 2015).

The PALOMA-1/TRIO-18 trial (Table 1 and Figure 3) was published in 2015 and represents the first and so far the only phase II study comparing letrozole monotherapy with the palbociclib/letrozole combination (Finn *et al*, 2015). The trial was an open-label, randomised study of postmenopausal women with advanced ER+/HER2− MBC. A total of 165 patients were enrolled in two separate cohorts: cohort 1 included 81 patients on the basis of their ER and HER2 status alone, whereas the 84 patients in cohort 2 were characterised by either amplification of cyclin D1 or loss of p16 or both. Patients were randomly assigned in both cohorts to receive continuous single-agent letrozole or the combination of letrozole and palbociclib. Overall, median PFS was 10.2 months in the letrozole monotherapy group that increased to 20.2 months in the combination group. In cohort 1, the PFS was 5.7 months in the letrozole monotherapy group and 26.1 months in the group treated with palbociclib in addition to letrozole. For cohort 2, the PFS was 11.1 months (letrozole monotherapy) and 18.1 months (combination), respectively. All in all, the study showed that patients with ER+/HER2− MBC had significantly longer PFS on combination therapy compared with single-agent letrozole. This finding was later on confirmed to be true for nearly all subgroups of patients (Finn *et al*, 2016). Currently, PALOMA-2, a phase III trial, is ongoing, resembling PALOMA-1 in a larger patient cohort. The final results are expected soon. Another ongoing phase III trial comparing palbociclib with exemestane or chemotherapy (capecitabine) in ER+/HER2− MBC patients with resistance to nonsteroidal AIs is currently recruiting.

In October 2016, Hortobagyi *et al* (2016) presented an early report covering a phase III clinical trial evaluating the efficacy of another CDK4/6 inhibitor, ribociclib, combined with letrozole *vs* letrozole given alone as systemic therapy in MBC patients. After 18 months, the PFS rate was 63% in the combination arm (ribociclib and letrozole) and 42.2% in the letrozole monotherapy arm. Although the PFS was significantly longer among those receiving ribociclib plus letrozole, this came at the cost of a higher rate of myelosuppression.

Additional trials involving other CDK4/6 inhibiting agents such as abemaciclib (LY2835219) and AIs in the metastatic setting are currently recruiting patients.

### Novel drug combinations involving AIs in MBC

It has been stated for several decades that combinations of traditional endocrine treatment options are not recommended for the treatment of ER+ MBC. Thus, combinations of, for example, antioestrogens and AIs, have not shown superiority compared with monotherapy in sequence. One of the major recently performed trials testing an AI (anastrozole) with an antioestrogen (tamoxifen) was the ATAC-trial in early BC (Cuzick *et al*, 2010). Again, the combination arm was terminated early because of lack of synergy and perhaps even detrimental effects. However, although early experience was based on clinical use of tamoxifen in drug combinations, the appearance of fulvestrant challenges this point of view. In contrast to tamoxifen, fulvestrant is a pure steroidal ER antagonist lacking oestrogen agonist effects. When bound to fulvestrant, the ER dimerisation is disrupted, making it unstable and thereby accelerating the degradation of the complex. Fulvestrant has been shown to be as least as efficient compared with AIs in the metastatic setting (Graham *et al*, 2016) when given in the optimal dose of 500 mg per month. Several studies combining fulvestrant with other endocrine agents have shown promising results (Massarweh *et al*, 2014; Cristofanilli *et al*, 2016; Ma *et al*, 2016). A few studies have tested dual endocrine treatment involving fulvestrant and an AI simultaneously. Mehta *et al* (2012) presented results that favoured the combination of fulvestrant and anastrozole *vs* anastrozole alone, and showed significant increase in both PFS and OS. Unfortunately, neither the FACT study (Bergh *et al*, 2012) nor the SoFEA trial (Johnston *et al*, 2013) could confirm that the combination of fulvestrant with either anastrozole or exemestane improves PFS compared with an AI alone. If subgroups of patients benefit from the combination of fulvestrant and an AI, these have to be defined in a better way before this strategy may be established as a standard.

Trials involving other compounds in combination with AIs included the farnesyl transferase inhibitors tipifarnib and lonafarnib (Johnston *et al*, 2008) and the antiangiogenetic agent bevacizumab (Yardley *et al*, 2011; Martin *et al*, 2015). One study showed increased PFS compared with previous trials involving hormonal therapy alone when combining bevacizumab with either anastrozole or fulvestrant (Yardley *et al*, 2011). Besides this study, none of the other combinations mentioned have showed a significant improvement in PFS or OS in patients with MBC.

The dual monoclonal antibody against IGFI/II MEDI-573 has showed preliminary activity in a heavily pretreated population of patients with different solid tumours (Haluska *et al*, 2014), and data from an ongoing study testing MEDI-573 in combination with AIs are expected next year. Another dual IGFI/insulin receptor inhibitor (BMS-754807) has also shown preclinical antitumour effect in combination with letrozole (Hou *et al*, 2011). A third agent targeting the IGF system (IGFRII) is ganitumab, an antibody that binds specifically to the IGFII receptor. It has shown an acceptable safety profile and preliminary antitumour response (Tolcher *et al*, 2009), but data published by Robertson *et al* (2013) comparing ganitumab with exemestane or fulvestrant showed that adding ganitumab did not improve outcomes.

Simultaneous blockade of both oestrogen and androgen biosynthesis has been suggested as another promising strategy to delay endocrine resistance during AI therapy. O'Shaughnessy *et al* (2016) recently reported a large phase II trial challenging this interesting hypothesis. The 297 ER+ MBC patients who were pretreated with a nonsteroidal AI were randomised (1 : 1 : 1) to receive oral once daily either 1000 mg abiraterone acetate (AA) plus 5 mg prednisone (arm A), 1000 mg abiraterone acetate plus 25 mg exemestane (arm B) or 25 mg exemestane monotherapy (arm C; O'Shaughnessy *et al*, 2016). Unfortunately, adding AA to exemestane did not improve PFS in this study. Additional studies testing antiandrogen therapies in combination with AIs are currently ongoing.

Finally, the histone deacetylase inhibitor entinostat showed acceptable safety profile and demonstrated clinical activity in ER+ MBC patients in a phase II study by Yardley *et al* (2013). A phase III trial investigating the clinical benefit of adding entinostat to exemestane in postmenopausal MBC patients is currently recruiting.

## Discussion

Following the establishment of AIs belonging to the third generation as first-line monotherapy in ER+ MBC, considerable efforts have been made to increase the duration of response by adding on other targeted therapies simultaneously. Based on crucial findings made in both basic and translational research, several mechanisms of adaptation to oestrogen depletion have been identified and targeted by novel drugs.

One of the first success stories was the co-administration of AIs and HER2 targeted drugs like trastuzumab and lapatinib in dual ER/HER2-positive subgroups of breast cancer patients. The crucial studies published by Johnston *et al* (2009) and Kaufman *et al* (2009) showed that adding either trastuzumab or lapatinib to an AI significantly increases PFS in ER+/HER2+ MBC patients. As a consequence, simultaneous therapy with AIs and anti-HER2 drugs is established as one potential treatment strategy in ER+/HER2+ patients. Following the identification of mTOR as another pivotal player in endocrine resistance, temsirolimus and everolimus were challenged in clinical trials as well. The crucial BOLERO-2 trial caused the worldwide breakthrough of mTOR inhibitors in addition to AIs. BOLERO-2 tested exemestane alone *vs* the combination with everolimus in postmenopausal patients progressing on a nonsteroidal AI (anastrozole or letrozole). The trial was highly positive, favouring the combination of exemestane and everolimus causing an increase in PFS of 6.5 months. Thus, exemestane combined with everolimus is recognised as well as one of the standard combination in clinical use for selected patients according to the design of the BOLERO-2 trial.

Motivated by the success stories following the combination of anti-HER2 and mTOR inhibitors with AIs, PIK3A inhibitors have entered the stage as well because of promising data obtained in early-phase studies (Bendell *et al*, 2012; Ando *et al*, 2014; Mayer *et al*, 2014; Rodon *et al*, 2014). However, the clinical role of PIK3A inhibitors in the future algorithms of MBC treatment remains to be clarified as we await the data from ongoing phase II and III trials. Finally, CDK4/6 inhibitors, influencing on the cell cycle itself, have shown promising results and are now tested in phase III trials. The phase II PALOMA-1/TRIO-18 presented an increase in PFS of 10 months when combining letrozole with palbociclib compared with letrozole alone (Finn *et al*, 2015). The phase III PALOMA-2 trial is ongoing, resembling PALOMA-1/TRIO-18 in a larger patient cohort. At this stage, palbociclib represents one of the most promising new agents suitable for combinations with AIs.

However, although several new classes of targeted therapies have shown some clinical efficacy when given simultaneously to AIs, endocrine resistance still occurs in all patients, although further delayed for a couple of months compared with AI monotherapy. Thus, additional translational cancer research is urgently needed to find new strategies that may delay tumour growth over longer time periods in ER+ breast cancer. Several new combinations have already been identified like combining AIs with pure antioestrogens or compounds interfering with the IGFR system.

Most importantly, from a clinical point of view, a significant increase in OS could not be demonstrated for the majority of drugs when combined with AIs. This may be because of the early clinical setting of AI drug combinations that are in general followed by a variety of antihormonal and chemotherapy lines. Whether some of the novel drug combinations involving AIs may transfer some time from the chemotherapy period of an individual patient into the on-treatment situation during endocrine therapy is not proven by hard evidence. On the other hand, the lack of influence on OS curves in general may also indicate that the additional blockade of an alternative signalling pathway may not cause a major breakthrough in breast cancer therapy (Tannock and Hickman, 2016) because of the networking of many parallel pathways and the plasticity of malignant cells in general.

The future impact of targeted therapy is further challenged by the emergence of completely novel strategies to treat advanced cancer like immunotherapy. The idea to combine several targeting drugs at the same time in patients is tempting, although increased toxicity has been reported in several trials involving 2–3 new compounds simultaneously. The side effects reported during novel drugs like cell cycle inhibiting drugs resemble partly the toxicity observed during classical chemotherapy (i.e., pancytopenia and so on). Thus, the major advantage of traditional antihormonal therapies like mild side effects and so on may be partly lost when initiating novel drug combinations. However, the introduction of novel screening methods like next-generation sequencing (NGS) will increase the ability to identify important targetable mutations in individual patients and hopefully pave the way for personalised drug combinations causing better clinical responses in selected patients.

In conclusion, we have recently seen significant improvements in PFS in selected breast cancer patients based on novel drug combinations involving AIs, mTOR inhibitors, HER2 directed therapies, PI3K inhibitors and CDK4/6 inhibitors. Unfortunately, no influence on OS in general indicates the urgent need of novel targets, improved patient selection based on novel biomarkers and, if possible, a better understanding of the complex adaptation processes underlying resistance to aromatase inhibition.

## Figures and Tables

**Table 1:** Pivotal clinical trials comparing AI monotherapy with drug combinations including AIs and novel, targeted drugs

**Study/phase**	**Patients**	***N***	**AI**	**Targeted drug**	**Efficacy/results**
**I. Studies combining AIs with HER1/2 inhibitors**					
EORTC 30008 Phase III, randomised, double-blind, placebo-controlled trial (Johnston *et al*, 2009)	PMW with MBC HR-pos, HER2-pos or -neg HR-pos/HER2-pos (subgroup) No prior ET for MBC was allowed.	1286 219	Letrozole	Lapatinib	Median PFS in the ER-pos/HER2-pos subgroup: 8.2 months (combination LAP+ LET) *vs* 3.0 months for LET monotherapy, HR 0.71, 95% CI: 0.53–0.96 (*P*=0.019).
TAnDEM study Phase III, randomised, open-label clinical trial (Kaufman *et al*, 2009)	PMW with HR-pos/HER2-pos MBC TAM or ANA as ET for MBC allowed for up to 4 weeks before inclusion.	207	Anastrozole	Trastuzumab	Median PFS: 4.8 months (combination ANA+TZM) *vs* 2.4 months for ANA monotherapy, HR=0.63, 95% CI: 0.47–0.84 (*P*=0.0016).
NCT00077025 Phase II, randomised placebo-controlled clinical trial (Cristofanilli *et al*, 2010)	PMW with HR-pos MBC; No prior ET for MBC.	94	Anastrozole	Gefitinib	Median PFS: 14.7 months in combination group (ANA+GEF) *vs* 8.4 months in the ANA+PLAC subgroup; HR: 0.55, CI: 0.32–0.94; CBR ANA+GEF: 49% CBR ANA+PLAC: 34%.
NCT00066378 Phase II randomised, double-blind, placebo-controlled trial (Tryfonidis *et al*, 2016)	PMW with HR-pos MBC LABC in subgroup of patients; Prior ET for MBC allowed.	71	Anastrozole	Gefitinib	PFS rate at 1 year: 35% for ANA+GEF *vs* 32% for ANA+PLAC; ORR: 22% ANA+GEF *vs* 28% ANA+PLAC; median duration of response: 13.8 months in the ANA+GEF group *vs* 18.6 months in the ANA+PLAC.
**II. Studies combining AIs with mTOR inhibitors**					
HORIZON Phase III, randomised, double-blind, placebo-controlled trial (Wolff *et al*, 2013)	PMW with HR-pos LABC or MBC; at least one measurable lesions by RECIST; no prior AI therapy for either LABC or MBC; no adjuvant AI therapy during the last 12 months before inclusion.	1112	Letrozole	Temsirolimus	Overall PFS was similar in both arms: 8.9 *vs* 9 months; HR: 0.90, 95% CI: 0.76–1.07 (*P*=0.25); PFS in patients ⩽age 65 years (exploratory analysis): 9.0 months in LET+TEM group *vs* 5.6 months in LET monotherapy arm; HR 0.75, 95% CI: 0.60–0.93 (*P*=0.009);
BOLERO-2 Phase III, randomised, double-blind, placebo-controlled trial (Baselga *et al*, 2012)	PMW with ER-pos/HER2-neg MBC refractory to previous LET or ANA monotherapy (recurrence during or within 12 months after the end of adjuvant therapy or within 1 month after the end of treatment for advanced disease.	724	Exemestane	Everolimus	Median PFS was 6.9 months with EXE+EVE and 2.8 months for EXE monotherapy; HR 0.43, CI 0.35–0.54 (*P*<0.001); Median central PFS: 10.6 (EXE+EVE) *vs* 4.1 months (EXE monotherapy); HR 0.36, 95% CI: 0.27–0.47 (*P*<0.001).
**III. Studies combining AIs with PI3K inhibitors**					
NCT01248494 Phase Ib, open-label clinical trial (Mayer *et al*, 2014)	PMW with ER-pos/HER2-neg MBC refractory to at least one line of ET in the MBC setting or diagnosed with MBC during or within 1 year of adjuvant ET.	51	Letrozole	Buparlisib	Clinical benefit rate at MTD of buparlisib: 31% 27% grade 3 adverse events. No grade 4 adverse events; Clinical efficacy not dependent on PIK3CA mutation status.
**IV. Studies combining AIs with CDK4/6 inhibitors**					
PALOMA-1/TRIO-18 Phase II, open-label, randomised trial (Finn *et al*, 2015)	PMW with advanced ER-pos/HER2-neg BC without prior treatment for advanced disease; Cohort 1: inclusion based on HR/HER2-status; Cohort 2: as cohort 1 but in addition confirmed amplification of cyclin D1, loss of p16 or both.	165	Letrozole	Palbociclib	Median PFS: 20.2 (LET+PAL) *vs* 10.2 months (LET monotherapy); HR 0.488, 95% CI: 0.319–0.748 (*P*=0.0004); Cohort 1: median PFS: 5.7 months (LET) *vs* 26.1 months for the LET+PAL combination; HR 0.299, 95% CI: 0.156–0.572 (one-sided *P*>0.0001); Cohort 2: median PFS: 11.1 months for LET monotherapy *vs* 18.1 months for the LET+PAL combination, HR 0.508, 95% CI: 0.303–0.853 (one-sided *P*=0.0046)
NCT01958021 Phase III, randomised, double-blind, placebo-controlled clinical trial (Hortobagyi *et al*, 2016)	PMW with HR-pos/HER2-neg recurrent or MBC without prior therapy for advanced breast cancer.	668	Letrozole	Ribociclib	PFS rate at 18 months: 63% for the LET+RIB combination *vs* 42.2% for the LET monotherapy arm, Overall response rate (patients with measureable disease at baseline): 52.7% for the LET+RIB combination *vs* 37.1% for the LET monotherapy group (*P*<0.001).
**V. Studies combining AIs with fulvestrant**					
FACT-trial Phase III, open-label, randomised, clinical trial (Bergh *et al*, 2012)	PMW or PREMPW receiving a GnRH agonist, with HR-pos MBC and relapse after or during primary treatment.	514	Anastrozole	Fulvestrant	Median TTP was 10.8 months for the ANA+FULV combination *vs* 10.2 months in the ANA monotherapy arm, HR 0.99, 95% CI: 0.81–1.20 (*P*=0.91); median overall survival was OS: 37.8 months and 38.2 months, respectively (*P*=1.00);
SoFEA Phase III, randomised, placebo-controlled clinical trial (Johnston *et al*, 2013)	PMW with HR-pos relapse or advanced BC (MBC or LABC) during therapy with an NSAI (NSAI given for at least 12 months as adjuvant therapy or at least 6 months for MBC).	723	Anastrozole Exemestane	Fulvestrant	PFS: ANA+FULV: 4.4 months (CI: 3.4–5.4 mo); FULV+PLAC: 4.8 months (CI: 3.6–5.5 mo); EXE mono: 3.4 months (CI: 3.0–4.6 mo); No difference was recorded comparing the ANA+FULV *vs* the FULV+PLAC groups or between the FULV+PLAC and EXE monotherapy groups.
NCT00075764 Phase III, randomised clinical trial (Mehta *et al*, 2012)	PMW with HR-pos MBC without prior therapy for MBC; prior adjuvant therapy with AI or TAM was allowed (following an early amendment).	707	Anastrozole	Fulvestrant	The median PFS was 13.5 months (95% CI: 12.1–15.1) for the ANA monotherapy arm *vs* 15.0 months (95% CI: 13.2–18.4) for the ANA+FULV arm (*P*=0.007); The median overall survival was 41.3 months (95% CI: 37.2–45.0) with ANA alone and 47.7 months (95% CI: 43.4–55.7) with ANA+FULV (*P*=0.049).
**VI. Studies combining AIs with alternative compounds**					
Exemestane±Etinostat trial Phase II, randomised, double-blind, placebo-controlled, ‘signal-finding' clinical trial (Yardley *et al*, 2013)	PMW with ER-pos BC experiencing relapse following adjuvant therapy with a NSAI (at least for 12 months) or progression of MBC during a NSAI (given for at least 3 months).	130	Exemestane	Etinostat	Median PFS was 4.3 months in the EXE+ETI group *vs* 2.3 months in the EXE+PLAC group; HR 0.73, 95% CI: 0.50–1.07 (*P*=0.055); Median overall survival was 28.1 months in the EXE+ETI arm *vs* 19.8 months in the EXE+PLAC subgroup, HR 0.59, 95% CI: 0.36–0.97 (*P*=0.036).
NCT00405938 Phase II, non-randomised clinical trial (Yardley *et al*, 2011)	PMW with HR-pos MBC without any previous therapy for MBC were eligible; HER2-pos and HER2neg patients could participate.	79	Anastrozole	Bevacizumab	Median TTP was 21 months for the combination ANA+BEV *vs* 9 months for the combination FULV+BEV; the overall response rates were 47% and 27%, respectively; both combinations were evaluated as feasible as first-line therapy for MBC and are currently tested in ongoing phase III trials.
LEA-study Phase III, randomised, open-label, clinical trial (Martin *et al*, 2015)	PMW with HR-pos/HER2-neg MBC; no prior therapy for MBC was allowed.	380	Letrozole or fulvestrant	Bevacizumab	Median PFS was 14.4 months in the ET-group (LET or FULV monotherapy) *vs* 19.3 months in the ET-B group (LET or FULV combined with BEV); HR 0.83, 95% CI: 0.65–1.06 (*P*=0.126); Overall response rate was 22% with ET *vs* 41% with ET-B, (*P*<0.001); TTF and OS were comparable in both arms.
NCT00050141 Phase II, randomised, blinded, parallel-group study (Johnston *et al*, 2008)	PMW with HR-pos BC progressing on tamoxifen either as adjuvant therapy or first line for advanced disease (objective response or stable disease during TAM-therapy was mandatory in MBC patients),	120	Letrozole	Tipifarnib	Median TTP was 10.8 months for the LET monotherapy arm *vs* 5.6 months for the combination LET+TIP; the ORR was 30% in the LET+TIP arm *vs* 38% in the LET monotherapy arm. Clinical benefit rate: 49% for the LET+TIP subgroup and 62% for the LET monotherapy cohort.

Abbreviations: AI=aromatase inhibitor; ANA=anastrozole; BC=breast cancer; CBR=clinical benefit rate; CI=confidence interval; ER-pos=oestrogen receptor positive; ET=endocrine treatment; ETI=etinostat; EVE=everolimus; FULV=fulvestrant; GEF=gefitinib; GnRH=gonadotropin-releasing hormone; HER2-pos=human epidermal growth factor receptor-2 positive; HR=hazard ratio; HR-pos=hormone receptor positive; LABC=locally advanced breast cancer; LAP=lapatinib; LET=letrozole; MBC=metastatic breast cancer; MTD=maximum tolerated dose; NSAI=nonsteroidal antiinflammatory (drugs); OLT=open-label trial; OS=overall survival; ORR=objective response rate; PFS=progression-free survival; PLAC=placebo; PMW=postmenopausal women; PREMPW=premenopausal women; RIB=ribociclib; TAM=tamoxifen; TEM=temsirolimus; TIP=tipifarnib; TTP=time to progression; TZM=trastuzumab.

## References

[bib1] Alberts B, Johnson A, Lewis J, Morgan D, Raff M, Roberts K, Walter P (2015) Molecular Biology of the Cell, 6th edn, Chapter 17. Taylor & Francis: Abingdon, UK, pp 967–1017.

[bib2] Ando Y, Inada-Inoue M, Mitsuma A, Yoshino T, Ohtsu A, Suenaga N, Sato M, Kakizume T, Robson M, Quadt C, Doi T (2014) Phase I dose-escalation study of buparlisib (BKM120), an oral pan-class I PI3K inhibitor, in Japanese patients with advanced solid tumors. Cancer Sci 105(3): 347–353. 24405565 10.1111/cas.12350 PMC4317947

[bib3] Baselga J, Campone M, Piccart M, Burris HA 3rd, Rugo HS, Sahmoud T, Noguchi S, Gnant M, Pritchard KI, Lebrun F, Beck JT, Ito Y, Yardley D, Deleu I, Perez A, Bachelot T, Vittori L, Xu Z, Mukhopadhyay P, Lebwohl D, Hortobagyi GN (2012) Everolimus in postmenopausal hormone-receptor-positive advanced breast cancer. N Engl J Med 366(6): 520–529. 22149876 10.1056/NEJMoa1109653 PMC5705195

[bib4] Beaver JA, Amiri-Kordestani L, Charlab R, Chen W, Palmby T, Tilley A, Zirkelbach JF, Yu J, Liu Q, Zhao L, Crich J, Chen XH, Hughes M, Bloomquist E, Tang S, Sridhara R, Kluetz PG, Kim G, Ibrahim A, Pazdur R, Cortazar P (2015) FDA approval: palbociclib for the treatment of postmenopausal patients with estrogen receptor-positive, HER2-negative metastatic breast cancer. Clin Cancer Res 21(21): 4760–4766. 26324739 10.1158/1078-0432.CCR-15-1185

[bib5] Bemanian V, Sauer T, Touma J, Lindstedt BA, Chen Y, Odegard HP, Vetvik KM, Bukholm IR, Geisler J (2015) The epidermal growth factor receptor (EGFR / HER-1) gatekeeper mutation T790M is present in European patients with early breast cancer. PLoS One 10(8): e0134398. 26267891 10.1371/journal.pone.0134398 PMC4534377

[bib6] Bendell JC, Rodon J, Burris HA, de Jonge M, Verweij J, Birle D, Demanse D, De Buck SS, Ru QC, Peters M, Goldbrunner M, Baselga J (2012) Phase I, dose-escalation study of BKM120, an oral pan-Class I PI3K inhibitor, in patients with advanced solid tumors. J Clin Oncol 30(3): 282–290. 22162589 10.1200/JCO.2011.36.1360

[bib7] Beresford M, Tumur I, Chakrabarti J, Barden J, Rao N, Makris A (2011) A qualitative systematic review of the evidence base for non-cross-resistance between steroidal and non-steroidal aromatase inhibitors in metastatic breast cancer. Clin Oncol 23(3): 209–215. 10.1016/j.clon.2010.11.005 21134732

[bib8] Bergh J, Jonsson PE, Lidbrink EK, Trudeau M, Eiermann W, Brattstrom D, Lindemann JP, Wiklund F, Henriksson R (2012) FACT: an open-label randomized phase III study of fulvestrant and anastrozole in combination compared with anastrozole alone as first-line therapy for patients with receptor-positive postmenopausal breast cancer. J Clin Oncol 30(16): 1919–1925. 22370325 10.1200/JCO.2011.38.1095

[bib9] Bertelli G, Garrone O, Merlano M, Occelli M, Bertolotti L, Castiglione F, Pepi F, Fusco O, Del Mastro L, Leonard RC (2005) Sequential treatment with exemestane and non-steroidal aromatase inhibitors in advanced breast cancer. Oncology 69(6): 471–477. 16410685 10.1159/000090985

[bib10] Blackwell K, Burris H, Gomez P, Lynn Henry N, Isakoff S, Campana F, Gao L, Jiang J, Mace S, Tolaney SM (2015) Phase I/II dose-escalation study of PI3K inhibitors pilaralisib or voxtalisib in combination with letrozole in patients with hormone-receptor-positive and HER2-negative metastatic breast cancer refractory to a non-steroidal aromatase inhibitor. Breast Cancer Res Treat 154(2): 287–297. 26497877 10.1007/s10549-015-3615-9

[bib11] Cadoo KA, Gucalp A, Traina TA (2014) Palbociclib: an evidence-based review of its potential in the treatment of breast cancer. Breast Cancer 6: 123–133. 25177151 10.2147/BCTT.S46725 PMC4128689

[bib12] Carey LA, Perou CM (2015) Palbociclib—taking breast-cancer cells out of gear. N Engl J Med 373(3): 273–274. 26176385 10.1056/NEJMe1506680

[bib13] Clarke R, Tyson JJ, Dixon JM (2015) Endocrine resistance in breast cancer—an overview and update. Mol Cell Endocrinol 418 Pt 3: 220–234. 26455641 10.1016/j.mce.2015.09.035 PMC4684757

[bib14] Cristofanilli M, Turner NC, Bondarenko I, Ro J, Im SA, Masuda N, Colleoni M, DeMichele A, Loi S, Verma S, Iwata H, Harbeck N, Zhang K, Theall KP, Jiang Y, Bartlett CH, Koehler M, Slamon D (2016) Fulvestrant plus palbociclib versus fulvestrant plus placebo for treatment of hormone-receptor-positive, HER2-negative metastatic breast cancer that progressed on previous endocrine therapy (PALOMA-3): final analysis of the multicentre, double-blind, phase 3 randomised controlled trial. Lancet Oncol 16(4): 425–439. 10.1016/S1470-2045(15)00613-0 26947331

[bib15] Cristofanilli M, Valero V, Mangalik A, Royce M, Rabinowitz I, Arena FP, Kroener JF, Curcio E, Watkins C, Bacus S, Cora EM, Anderson E, Magill PJ (2010) Phase II, randomized trial to compare anastrozole combined with gefitinib or placebo in postmenopausal women with hormone receptor-positive metastatic breast cancer. Clin Cancer Res 16(6): 1904–1914. 20215537 10.1158/1078-0432.CCR-09-2282

[bib16] Curley MD, Sabnis GJ, Wille L, Adiwijaya BS, Garcia G, Moyo V, Kazi AA, Brodie A, MacBeath G (2015) Seribantumab, an anti-ERBB3 antibody, delays the onset of resistance and restores sensitivity to letrozole in an estrogen receptor-positive breast cancer model. Mol Cancer Ther 14(11): 2642–2652. 26310543 10.1158/1535-7163.MCT-15-0169

[bib17] Cuzick J, Sestak I, Baum M, Buzdar A, Howell A, Dowsett M, Forbes JF (2010) Effect of anastrozole and tamoxifen as adjuvant treatment for early-stage breast cancer: 10-year analysis of the ATAC trial. Lancet Oncol 11(12): 1135–1141. 21087898 10.1016/S1470-2045(10)70257-6

[bib18] Dickson MA (2014) Molecular pathways: CDK4 inhibitors for cancer therapy. Clin Cancer Res 20(13): 3379–3383. 24795392 10.1158/1078-0432.CCR-13-1551

[bib19] Dowsett M, Martin LA, Smith I, Johnston S (2005) Mechanisms of resistance to aromatase inhibitors. J Steroid Biochem Mol Biol 95(1-5): 167–172. 15982868 10.1016/j.jsbmb.2005.04.022

[bib20] Elebro K, Borgquist S, Simonsson M, Markkula A, Jirstrom K, Ingvar C, Rose C, Jernstrom H (2015) Combined androgen and estrogen receptor status in breast cancer: treatment prediction and prognosis in a population-based prospective cohort. Clin Cancer Res 21(16): 3640–3650. 25904752 10.1158/1078-0432.CCR-14-2564

[bib21] Fan W, Chang J, Fu P (2015) Endocrine therapy resistance in breast cancer: current status, possible mechanisms and overcoming strategies. Future Med Chem 7(12): 1511–1519. 26306654 10.4155/fmc.15.93 PMC5558537

[bib22] Finn RS, Crown JP, Ettl J, Schmidt M, Bondarenko IM, Lang I, Pinter T, Boer K, Patel R, Randolph S, Kim ST, Huang X, Schnell P, Nadanaciva S, Bartlett CH, Slamon DJ (2016) Efficacy and safety of palbociclib in combination with letrozole as first-line treatment of ER-positive, HER2-negative, advanced breast cancer: expanded analyses of subgroups from the randomized pivotal trial PALOMA-1/TRIO-18. Breast Cancer Res 18(1): 67. 27349747 10.1186/s13058-016-0721-5 PMC4924326

[bib23] Finn RS, Crown JP, Lang I, Boer K, Bondarenko IM, Kulyk SO, Ettl J, Patel R, Pinter T, Schmidt M, Shparyk Y, Thummala AR, Voytko NL, Fowst C, Huang X, Kim ST, Randolph S, Slamon DJ (2015) The cyclin-dependent kinase 4/6 inhibitor palbociclib in combination with letrozole versus letrozole alone as first-line treatment of oestrogen receptor-positive, HER2-negative, advanced breast cancer (PALOMA-1/TRIO-18): a randomised phase 2 study. Lancet Oncol 16(1): 25–35. 25524798 10.1016/S1470-2045(14)71159-3

[bib24] Flågeng MH, Moi LL, Dixon JM, Geisler J, Lien EA, Miller WR, Lønning PE, Mellgren G (2009) Nuclear receptor co-activators and HER-2/neu are upregulated in breast cancer patients during neo-adjuvant treatment with aromatase inhibitors. Br J Cancer 101(8): 1253–1260. 19755984 10.1038/sj.bjc.6605324 PMC2768454

[bib25] Fujii R, Hanamura T, Suzuki T, Gohno T, Shibahara Y, Niwa T, Yamaguchi Y, Ohnuki K, Kakugawa Y, Hirakawa H, Ishida T, Sasano H, Ohuchi N, Hayashi S (2014) Increased androgen receptor activity and cell proliferation in aromatase inhibitor-resistant breast carcinoma. J Steroid Biochem Mol Biol 144: 513–522. 25178713 10.1016/j.jsbmb.2014.08.019

[bib26] Gee JM, Robertson JF, Gutteridge E, Ellis IO, Pinder SE, Rubini M, Nicholson RI (2005) Epidermal growth factor receptor/HER2/insulin-like growth factor receptor signalling and oestrogen receptor activity in clinical breast cancer. Endocr Relat Cancer 12(Suppl 1): S99–s111. 16113104 10.1677/erc.1.01005

[bib27] Geisler J (2003) Aromatase inhibitors and inactivators for the treatment of postmenopausal breast cancer: a review. Curr Med Chem 3: 261–276.

[bib28] Geisler J, Lønning PE (2005) Aromatase inhibition: translation into a successful therapeutic approach. Clin Cancer Res 11(8): 2809–2821. 15837728 10.1158/1078-0432.CCR-04-2187

[bib29] Geuna E, Milani A, Martinello R, Aversa C, Valabrega G, Scaltriti M, Montemurro F (2015) Buparlisib, an oral pan-PI3K inhibitor for the treatment of breast cancer. Expert Opin Investig Drugs 24(3): 421–431. 10.1517/13543784.2015.1008132 25645727

[bib30] Gibson L, Lawrence D, Dawson C, Bliss J (2009) Aromatase inhibitors for treatment of advanced breast cancer in postmenopausal women. Cochrane Database Syst Rev 4: CD003370. 10.1002/14651858.CD003370.pub3 PMC7154337 19821307

[bib31] Graham J, Pitz M, Gordon V, Grenier D, Amir E, Niraula S (2016) Clinical predictors of benefit from fulvestrant in advanced breast cancer: a meta-analysis of randomized controlled trials. Cancer Treat Rev 45: 1–6. 26922660 10.1016/j.ctrv.2016.02.004

[bib32] Haluska P, Menefee M, Plimack ER, Rosenberg J, Northfelt D, LaVallee T, Shi L, Yu XQ, Burke P, Huang J, Viner J, McDevitt J, LoRusso P (2014) Phase I dose-escalation study of MEDI-573, a bispecific, antiligand monoclonal antibody against IGFI and IGFII, in patients with advanced solid tumors. Clin Cancer Res 20(18): 4747–4757. 25024259 10.1158/1078-0432.CCR-14-0114 PMC4377301

[bib33] Hayes EL, Lewis-Wambi JS (2015) Mechanisms of endocrine resistance in breast cancer: an overview of the proposed roles of noncoding RNA. Breast Cancer Res 17: 40. 25849966 10.1186/s13058-015-0542-y PMC4362832

[bib34] Hong Y, Chen S (2006) Aromatase inhibitors: structural features and biochemical characterization. Ann NY Acad Sci 1089: 237–251. 17261771 10.1196/annals.1386.022

[bib35] Hortobagyi GN, Stemmer SM, Burris HA, Yap YS, Sonke GS, Paluch-Shimon S, Campone M, Blackwell KL, Andre F, Winer EP, Janni W, Verma S, Conte P, Arteaga CL, Cameron DA, Petrakova K, Hart LL, Villanueva C, Chan A, Jakobsen E, Nusch A, Burdaeva O, Grischke EM, Alba E, Wist E, Marschner N, Favret AM, Yardley D, Bachelot T, Tseng LM, Blau S, Xuan F, Souami F, Miller M, Germa C, Hirawat S, O'Shaughnessy J (2016) Ribociclib as first-line therapy for HR-positive, advanced breast cancer. N Engl J Med 375(18): 1738–1748. 27717303 10.1056/NEJMoa1609709

[bib36] Hou X, Huang F, Macedo LF, Harrington SC, Reeves KA, Greer A, Finckenstein FG, Brodie A, Gottardis MM, Carboni JM, Haluska P (2011) Dual IGF-1R/InsR inhibitor BMS-754807 synergizes with hormonal agents in treatment of estrogen-dependent breast cancer. Cancer Res 71(24): 7597–7607. 22042792 10.1158/0008-5472.CAN-11-1080 PMC4004036

[bib37] James A, Blumenstein L, Glaenzel U, Jin Y, Demailly A, Jakab A, Hansen R, Hazell K, Mehta A, Trandafir L, Swart P (2015) Absorption, distribution, metabolism, and excretion of [(14)C]BYL719 (alpelisib) in healthy male volunteers. Cancer Chem Pharm 76(4): 751–760. 10.1007/s00280-015-2842-4 26254025

[bib38] Johnston S, Pippen J Jr, Pivot X, Lichinitser M, Sadeghi S, Dieras V, Gomez HL, Romieu G, Manikhas A, Kennedy MJ, Press MF, Maltzman J, Florance A, O'Rourke L, Oliva C, Stein S, Pegram M (2009) Lapatinib combined with letrozole versus letrozole and placebo as first-line therapy for postmenopausal hormone receptor-positive metastatic breast cancer. J Clin Oncol 27(33): 5538–5546. 19786658 10.1200/JCO.2009.23.3734

[bib39] Johnston SR, Kilburn LS, Ellis P, Dodwell D, Cameron D, Hayward L, Im YH, Braybrooke JP, Brunt AM, Cheung KL, Jyothirmayi R, Robinson A, Wardley AM, Wheatley D, Howell A, Coombes G, Sergenson N, Sin HJ, Folkerd E, Dowsett M, Bliss JM (2013) Fulvestrant plus anastrozole or placebo versus exemestane alone after progression on non-steroidal aromatase inhibitors in postmenopausal patients with hormone-receptor-positive locally advanced or metastatic breast cancer (SoFEA): a composite, multicentre, phase 3 randomised trial. Lancet Oncol 14(10): 989–998. 23902874 10.1016/S1470-2045(13)70322-X

[bib40] Johnston SR, Semiglazov VF, Manikhas GM, Spaeth D, Romieu G, Dodwell DJ, Wardley AM, Neven P, Bessems A, Park YC, De Porre PM, Perez Ruixo JJ, Howes AJ (2008) A phase II, randomized, blinded study of the farnesyltransferase inhibitor tipifarnib combined with letrozole in the treatment of advanced breast cancer after antiestrogen therapy. Breast Cancer Res Treat 110(2): 327–335. 17851757 10.1007/s10549-007-9726-1

[bib41] Kaufman B, Mackey JR, Clemens MR, Bapsy PP, Vaid A, Wardley A, Tjulandin S, Jahn M, Lehle M, Feyereislova A, Revil C, Jones A (2009) Trastuzumab plus anastrozole versus anastrozole alone for the treatment of postmenopausal women with human epidermal growth factor receptor 2-positive, hormone receptor-positive metastatic breast cancer: results from the randomized phase III TAnDEM study. J Clin Oncol 27(33): 5529–5537. 19786670 10.1200/JCO.2008.20.6847

[bib42] Klumpen HJ, Beijnen JH, Gurney H, Schellens JH (2010) Inhibitors of mTOR. Oncologist 15(12): 1262–1269. 21147869 10.1634/theoncologist.2010-0196 PMC3227930

[bib43] Leary A, Dowsett M (2006) Combination therapy with aromatase inhibitors: the next era of breast cancer treatment? Br J Cancer 95(6): 661–666. 16926831 10.1038/sj.bjc.6603316 PMC2360507

[bib44] Liu C, Zhang Z, Tang H, Jiang Z, You L, Liao Y (2014) Crosstalk between IGF-1R and other tumor promoting pathways. Curr Pharm Des 20(17): 2912–2921. 23944361 10.2174/13816128113199990596

[bib45] Liu N, Rowley BR, Bull CO, Schneider C, Haegebarth A, Schatz CA, Fracasso PR, Wilkie DP, Hentemann M, Wilhelm SM, Scott WJ, Mumberg D, Ziegelbauer K (2013) BAY 80-6946 is a highly selective intravenous PI3K inhibitor with potent p110alpha and p110delta activities in tumor cell lines and xenograft models. Mol Cancer Ther 12(11): 2319–2330. 24170767 10.1158/1535-7163.MCT-12-0993-T

[bib46] Lønning PE (2009) Lack of complete cross-resistance between different aromatase inhibitors; a real finding in search for an explanation? Eur J Cancer 45(4): 527–535. 19062270 10.1016/j.ejca.2008.10.019

[bib47] Lopez-Knowles E, Segal CV, Gao Q, Garcia-Murillas I, Turner NC, Smith I, Martin LA, Dowsett M (2014) Relationship of PIK3CA mutation and pathway activity with antiproliferative response to aromatase inhibition. Breast Cancer Res 16(3): R68. 24981670 10.1186/bcr3683 PMC4227109

[bib48] LoRusso PM (2013) Mammalian target of rapamycin as a rational therapeutic target for breast cancer treatment. Oncology 84(1): 43–56. 23128843 10.1159/000343063

[bib49] Lupien M, Meyer CA, Bailey ST, Eeckhoute J, Cook J, Westerling T, Zhang X, Carroll JS, Rhodes DR, Liu XS, Brown M (2010) Growth factor stimulation induces a distinct ER(alpha) cistrome underlying breast cancer endocrine resistance. Genes Dev 24(19): 2219–2227. 20889718 10.1101/gad.1944810 PMC2947773

[bib50] Ma CX, Luo J, Naughton M, Ademuyiwa F, Suresh R, Griffith M, Griffith OL, Skidmore ZL, Spies NC, Ramu A, Trani L, Pluard T, Nagaraj G, Thomas S, Guo Z, Hoog J, Han J, Mardis E, Lockhart C, Ellis MJ (2016) A Phase I trial of BKM120 (buparlisib) in combination with fulvestrant in postmenopausal women with estrogen receptor-positive metastatic breast cancer. Clin Cancer Res 22(7): 1583–1591. 26563128 10.1158/1078-0432.CCR-15-1745 PMC4818722

[bib51] Ma CX, Reinert T, Chmielewska I, Ellis MJ (2015) Mechanisms of aromatase inhibitor resistance. Nat Rev Cancer 15(5): 261–275. 25907219 10.1038/nrc3920

[bib52] Martin M, Loibl S, von Minckwitz G, Morales S, Martinez N, Guerrero A, Anton A, Aktas B, Schoenegg W, Munoz M, Garcia-Saenz JA, Gil M, Ramos M, Margeli M, Carrasco E, Liedtke C, Wachsmann G, Mehta K, De la Haba-Rodriguez JR (2015) Phase III trial evaluating the addition of bevacizumab to endocrine therapy as first-line treatment for advanced breast cancer: the letrozole/fulvestrant and avastin (LEA) study. J Clin Oncol 33(9): 1045–1052. 25691671 10.1200/JCO.2014.57.2388

[bib53] Massacesi C, Di Tomaso E, Urban P, Germa C, Quadt C, Trandafir L, Aimone P, Fretault N, Dharan B, Tavorath R, Hirawat S (2016) PI3K inhibitors as new cancer therapeutics: implications for clinical trial design. Onco Targets Ther 9: 203–210. 26793003 10.2147/OTT.S89967 PMC4708174

[bib54] Massarweh S, Romond E, Black EP, Van Meter E, Shelton B, Kadamyan-Melkumian V, Stevens M, Elledge R (2014) A phase II study of combined fulvestrant and everolimus in patients with metastatic estrogen receptor (ER)-positive breast cancer after aromatase inhibitor (AI) failure. Breast Cancer Res Treat 143(2): 325–332. 24327334 10.1007/s10549-013-2810-9

[bib55] Mayer IA, Abramson VG, Isakoff SJ, Forero A, Balko JM, Kuba MG, Sanders ME, Yap JT, Van den Abbeele AD, Li Y, Cantley LC, Winer E, Arteaga CL (2014) Stand up to cancer phase Ib study of pan-phosphoinositide-3-kinase inhibitor buparlisib with letrozole in estrogen receptor-positive/human epidermal growth factor receptor 2-negative metastatic breast cancer. J Clin Oncol 32(12): 1202–1209. 24663045 10.1200/JCO.2013.54.0518 PMC3986383

[bib56] Mehta RS, Barlow WE, Albain KS, Vandenberg TA, Dakhil SR, Tirumali NR, Lew DL, Hayes DF, Gralow JR, Livingston RB, Hortobagyi GN (2012) Combination anastrozole and fulvestrant in metastatic breast cancer. N Engl J Med 367(5): 435–444. 22853014 10.1056/NEJMoa1201622 PMC3951300

[bib57] Miller TW, Balko JM, Arteaga CL (2011) Phosphatidylinositol 3-kinase and antiestrogen resistance in breast cancer. J Clin Oncol 29(33): 4452–4461. 22010023 10.1200/JCO.2010.34.4879 PMC3221526

[bib58] Ndubaku CO, Heffron TP, Staben ST, Baumgardner M, Blaquiere N, Bradley E, Bull R, Do S, Dotson J, Dudley D, Edgar KA, Friedman LS, Goldsmith R, Heald RA, Kolesnikov A, Lee L, Lewis C, Nannini M, Nonomiya J, Pang J, Price S, Prior WW, Salphati L, Sideris S, Wallin JJ, Wang L, Wei B, Sampath D, Olivero AG (2013) Discovery of 2-{3-[2-(1-isopropyl-3-methyl-1H-1,2-4-triazol-5-yl)-5,6-dihydrobenzo[f]imidazo[1,2-d][1,4]oxazepin-9-yl]-1H-pyrazol-1-yl}-2-methylpropanamide (GDC-0032): a beta-sparing phosphoinositide 3-kinase inhibitor with high unbound exposure and robust *in vivo* antitumor activity. J Med Chem 56(11): 4597–4610. 23662903 10.1021/jm4003632

[bib59] O'Shaughnessy J, Campone M, Brain E, Neven P, Hayes D, Bondarenko I, Griffin TW, Martin J, De Porre P, Kheoh T, Yu MK, Peng W, Johnston S (2016) Abiraterone acetate, exemestane or the combination in postmenopausal patients with estrogen receptor-positive metastatic breast cancer. Ann Oncol 27(1): 106–113. 26504153 10.1093/annonc/mdv487 PMC4684153

[bib60] Park S, Koo J, Park HS, Kim JH, Choi SY, Lee JH, Park BW, Lee KS (2010) Expression of androgen receptors in primary breast cancer. Ann Oncol 21(3): 488–492. 19887463 10.1093/annonc/mdp510

[bib61] Paul B, Trovato JA, Thompson J (2008) Lapatinib: a dual tyrosine kinase inhibitor for metastatic breast cancer. Am J Health Syst Pharm 65(18): 1703–1710. 18768996 10.2146/ajhp070646

[bib62] Piccart M, Hortobagyi GN, Campone M, Pritchard KI, Lebrun F, Ito Y, Noguchi S, Perez A, Rugo HS, Deleu I, Burris HA 3rd, Provencher L, Neven P, Gnant M, Shtivelband M, Wu C, Fan J, Feng W, Taran T, Baselga J (2014) Everolimus plus exemestane for hormone-receptor-positive, human epidermal growth factor receptor-2-negative advanced breast cancer: overall survival results from BOLERO-2dagger. Ann Oncol 25(12): 2357–2362. 25231953 10.1093/annonc/mdu456 PMC6267855

[bib63] Rechoum Y, Rovito D, Iacopetta D, Barone I, Ando S, Weigel NL, O'Malley BW, Brown PH, Fuqua SA (2014) AR collaborates with ERalpha in aromatase inhibitor-resistant breast cancer. Breast Cancer Res Treat 147(3): 473–485. 25178514 10.1007/s10549-014-3082-8 PMC4337991

[bib64] Riemsma R, Forbes CA, Amonkar MM, Lykopoulos K, Diaz JR, Kleijnen J, Rea DW (2012) Systematic review of lapatinib in combination with letrozole compared with other first-line treatments for hormone receptor positive(HR+) and HER2+ advanced or metastatic breast cancer(MBC). Curr Med Res Opin 28(8): 1263–1279. 22738819 10.1185/03007995.2012.707643

[bib65] Robertson JF, Ferrero JM, Bourgeois H, Kennecke H, de Boer RH, Jacot W, McGreivy J, Suzuki S, Zhu M, McCaffery I, Loh E, Gansert JL, Kaufman PA (2013) Ganitumab with either exemestane or fulvestrant for postmenopausal women with advanced, hormone-receptor-positive breast cancer: a randomised, controlled, double-blind, phase 2 trial. Lancet Oncol 14(3): 228–235. 23414585 10.1016/S1470-2045(13)70026-3

[bib66] Rocca A, Farolfi A, Bravaccini S, Schirone A, Amadori D (2014) Palbociclib (PD 0332991): targeting the cell cycle machinery in breast cancer. Exp Opin Pharm 15(3): 407–420. 10.1517/14656566.2014.870555 24369047

[bib67] Rodon J, Brana I, Siu LL, De Jonge MJ, Homji N, Mills D, Di Tomaso E, Sarr C, Trandafir L, Massacesi C, Eskens F, Bendell JC (2014) Phase I dose-escalation and -expansion study of buparlisib (BKM120), an oral pan-Class I PI3K inhibitor, in patients with advanced solid tumors. Invest New Drugs 32(4): 670–681. 24652201 10.1007/s10637-014-0082-9

[bib68] Sanchez CG, Ma CX, Crowder RJ, Guintoli T, Phommaly C, Gao F, Lin L, Ellis MJ (2011) Preclinical modeling of combined phosphatidylinositol-3-kinase inhibition with endocrine therapy for estrogen receptor-positive breast cancer. Breast Cancer Res 13(2): R21. 21362200 10.1186/bcr2833 PMC3219179

[bib69] Sarker D, Ang JE, Baird R, Kristeleit R, Shah K, Moreno V, Clarke PA, Raynaud FI, Levy G, Ware JA, Mazina K, Lin R, Wu J, Fredrickson J, Spoerke JM, Lackner MR, Yan Y, Friedman LS, Kaye SB, Derynck MK, Workman P, de Bono JS (2015) First-in-human phase I study of pictilisib (GDC-0941), a potent pan-class I phosphatidylinositol-3-kinase (PI3K) inhibitor, in patients with advanced solid tumors. Clin Cancer Res 21(1): 77–86. 25370471 10.1158/1078-0432.CCR-14-0947 PMC4287394

[bib70] Schwartzberg LS, Franco SX, Florance A, O'Rourke L, Maltzman J, Johnston S (2010) Lapatinib plus letrozole as first-line therapy for HER-2+ hormone receptor-positive metastatic breast cancer. Oncologist 15(2): 122–129. 20156908 10.1634/theoncologist.2009-0240 PMC3227947

[bib71] Tannock IF, Hickman JA (2016) Limits to personalized cancer medicine. N Engl J Med 375(13): 1289–1294. 27682039 10.1056/NEJMsb1607705

[bib72] Thomas C, Gustafsson JA (2015) Estrogen receptor mutations and functional consequences for breast cancer. Trends Endocrinol Metab 26(9): 467–476. 26183887 10.1016/j.tem.2015.06.007

[bib73] Tolcher AW, Sarantopoulos J, Patnaik A, Papadopoulos K, Lin CC, Rodon J, Murphy B, Roth B, McCaffery I, Gorski KS, Kaiser B, Zhu M, Deng H, Friberg G, Puzanov I (2009) Phase I, pharmacokinetic, and pharmacodynamic study of AMG 479, a fully human monoclonal antibody to insulin-like growth factor receptor 1. J Clin Oncol 27(34): 5800–5807. 19786654 10.1200/JCO.2009.23.6745

[bib74] Tryfonidis K, Basaran G, Bogaerts J, Debled M, Dirix L, Thery JC, Tjan-Heijnen VC, Van den Weyngaert D, Cufer T, Piccart M, Cameron D (2016) A European Organisation for Research and Treatment of Cancer randomized, double-blind, placebo-controlled, multicentre phase II trial of anastrozole in combination with gefitinib or placebo in hormone receptor-positive advanced breast cancer (NCT00066378). Eur J Cancer 53: 144–154. 26724641 10.1016/j.ejca.2015.10.012

[bib75] Van Asten K, Neven P, Lintermans A, Wildiers H, Paridaens R (2014) Aromatase inhibitors in the breast cancer clinic: focus on exemestane. Endocr Relat Cancer 21(1): R31–R49. 24434719 10.1530/ERC-13-0269

[bib76] Vidula N, Rugo HS (2016) Cyclin-dependent kinase 4/6 inhibitors for the treatment of breast cancer: a review of preclinical and clinical data. Clin Breast Cancer 16(1): 8–17. 26303211 10.1016/j.clbc.2015.07.005

[bib77] Wang X, Ding J, Meng LH (2015) PI3K isoform-selective inhibitors: next-generation targeted cancer therapies. Acta Pharmacol Sin 36(10): 1170–1176. 26364801 10.1038/aps.2015.71 PMC4648175

[bib78] Witkiewicz AK, Knudsen ES (2014) Retinoblastoma tumor suppressor pathway in breast cancer: prognosis, precision medicine, and therapeutic interventions. Breast Cancer Res 16(3): 207. 25223380 10.1186/bcr3652 PMC4076637

[bib79] Wolff AC, Lazar AA, Bondarenko I, Garin AM, Brincat S, Chow L, Sun Y, Neskovic-Konstantinovic Z, Guimaraes RC, Fumoleau P, Chan A, Hachemi S, Strahs A, Cincotta M, Berkenblit A, Krygowski M, Kang LL, Moore L, Hayes DF (2013) Randomized phase III placebo-controlled trial of letrozole plus oral temsirolimus as first-line endocrine therapy in postmenopausal women with locally advanced or metastatic breast cancer. J Clin Oncol 31(2): 195–202. 23233719 10.1200/JCO.2011.38.3331 PMC3532391

[bib80] Yardley DA, Burris HA 3rd, Clark BL, Shipley D, Rubin M, Barton J Jr, Arrowsmith E, Hainsworth JD (2011) Hormonal therapy plus bevacizumab in postmenopausal patients who have hormone receptor-positive metastatic breast cancer: a phase II Trial of the Sarah Cannon Oncology Research Consortium. Clin Breast Cancer 11(3): 146–152. 21665134 10.1016/j.clbc.2011.03.010

[bib81] Yardley DA, Ismail-Khan RR, Melichar B, Lichinitser M, Munster PN, Klein PM, Cruickshank S, Miller KD, Lee MJ, Trepel JB (2013) Randomized phase II, double-blind, placebo-controlled study of exemestane with or without entinostat in postmenopausal women with locally recurrent or metastatic estrogen receptor-positive breast cancer progressing on treatment with a nonsteroidal aromatase inhibitor. J Clin Oncol 31(17): 2128–2135. 23650416 10.1200/JCO.2012.43.7251 PMC4881332

[bib82] Yates LR, Gerstung M, Knappskog S, Desmedt C, Gundem G, Van Loo P, Aas T, Alexandrov LB, Larsimont D, Davies H, Li Y, Ju YS, Ramakrishna M, Haugland HK, Lilleng PK, Nik-Zainal S, McLaren S, Butler A, Martin S, Glodzik D, Menzies A, Raine K, Hinton J, Jones D, Mudie LJ, Jiang B, Vincent D, Greene-Colozzi A, Adnet PY, Fatima A, Maetens M, Ignatiadis M, Stratton MR, Sotiriou C, Richardson AL, Lønning PE, Wedge DC, Campbell PJ (2015) Subclonal diversification of primary breast cancer revealed by multiregion sequencing. Nat Med 21(7): 751–759. 26099045 10.1038/nm.3886 PMC4500826

